# Impact of COVID‑19 infection on subsequent prescriptions of autonomic dysfunction pharmacotherapy: a nationwide propensity‑score‑matched Cohort study in Japan

**DOI:** 10.1080/07853890.2026.2618323

**Published:** 2026-01-20

**Authors:** Daisuke Miyamori, Masanori Ito

**Affiliations:** Department of General Internal Medicine, Hiroshima University Hospital, Hiroshima, Japan

**Keywords:** COVID-19, autonomic dysfunction, midodrine, fludrocortisone, droxidopa

## Abstract

**Background:**

Autonomic dysfunction, including orthostatic hypotension and postural tachycardia syndrome, has emerged as a COVID-19 complication. This nationwide propensity score-matched cohort study investigated COVID-19’s impact on subsequent prescriptions of autonomic dysfunction in Japan.

**Patients and methods:**

Using a claims database covering 16 million residents identified between 2020 and 2022, propensity-score matching (PSM) created comparable groups of COVID-19 patients and controls. PSM used age, sex, calendar month, comorbidities, and baseline medications, with nearest-neighbor 1:1 with replacement. The primary composite outcome was the first outpatient prescription of midodrine, fludrocortisone, amezinium methylsulfate, and droxidopa. Cox proportional hazards models yielded hazard ratios (HRs) with 95% confidence intervals (CIs). Effect modifications were examined by subgroups.

**Results:**

Among 3,074,329 matched pairs, over a median follow-up of 8 months, 13011 composite outcome were observed, and COVID-19 infection was associated with a 36% relative increase in prescriptions (HR 1.36, 95%CI 1.32–1.41). The risk persisted beyond one year, with the strongest association observed for fludrocortisone (576 events, HR 1.71, 95%CI 1.44–2.02), although the frequency was the highest in midodrine prescription (7009 events, HR 1.28, 95%CI 1.22–1.34). Subgroup analysis revealed higher risks among older individuals, males, those with myocardial infarction, heart failure, and antihypertensive medications.

**Conclusions:**

COVID-19 infection is significantly associated with increased initiation of pharmacotherapy for autonomic dysfunction, with sustained risk beyond one year. These findings highlight the to manage autonomic dysfunction among COVID-19 survivors and informing clinical care and public health planning.

## Graphical Abstract

**Figure:**
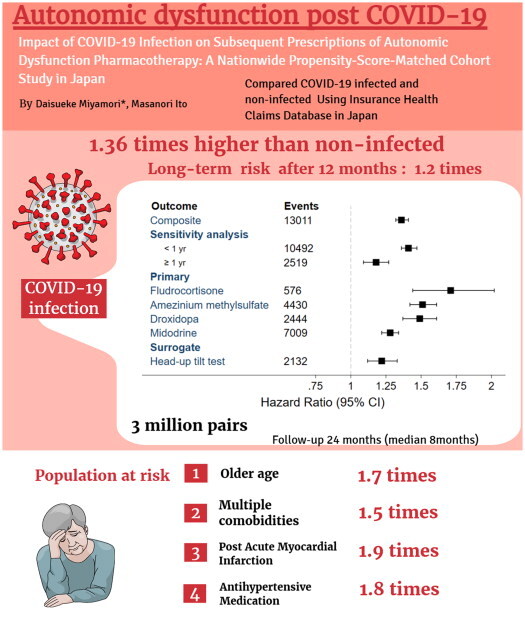

## Introduction

The emergence of the coronavirus disease 2019 (COVID-19) has dramatically altered the landscape of autonomic dysfunction. Since early in the pandemic, numerous case series and observational studies have reported that ‘long COVID’ is frequently complicated by persistent autonomic symptoms such as palpitations, syncope, and disabling fatigue, which can persist for months beyond acute infection [1]. Mechanistic research has suggested that immune-mediated ganglionic injury and persistent viral reservoirs in the brainstem may contribute to these syndromes [2]. Population-level data from multiple countries now indicate a marked rise in the incidence of post-acute sequelae of COVID-19, including new-onset autonomic dysfunction [3]. However, the clinical implications of this increase, particularly how it affects real-world prescription and pharmacotherapy needs, remain unclear.

Autonomic dysfunction, including orthostatic hypotension, postural tachycardia syndrome (POTS), and other neurocardiogenic disorders, represents a significant and growing challenge in clinical practice. Traditionally, the management of these conditions has relied on pharmacotherapy with agents such as midodrine, fludrocortisone, amezinium methylsulfate, and droxidopa, often after non-pharmacological measures fail [4,5]. However, even before the COVID-19 pandemic, treatment strategies remained suboptimal, and high-quality evidence to guide clinical care is limited.

Understanding the impact of COVID-19 on pharmacological management of autonomic dysfunction is of urgent clinical importance. First, the sheer scale of the pandemic has resulted in an unprecedented number of patients being at risk for chronic autonomic complications. Second, evidence supporting the efficacy or appropriateness of pharmacotherapy for post-COVID autonomic dysfunction (PASC-AD) is extremely limited. Third, the unique and potentially severe pathophysiological mechanisms induced by COVID-19, including autoimmunity, microvascular injury, and direct neuronal damage, may render existing treatment paradigms less effective and create new patterns of medication utilization. This has direct implications for healthcare resource allocation, patient quality of life, and long-term morbidities.

Despite the recognition of the association between COVID-19 and autonomic dysfunction, there is a critical gap in our understanding of how this translates to changes in pharmacotherapy practice. The 2024 American Heart Association (AHA) scientific statement specifically calls for large-scale, real-world data to inform treatment strategies for post-COVID autonomic disorders [6]. Most prior studies have been limited by a single-center design, lack of control groups, and absence of population-level pharmaco-epidemiological data.

To address this gap, we conducted a nationwide propensity-score-matched cohort study using a comprehensive Japanese claims database. Our objectives were (i) to determine whether COVID-19 infection increases the subsequent initiation of pharmacotherapy for autonomic dysfunction, (ii) to describe absolute and relative risks over time, and (iii) to identify which patient subgroups are most vulnerable to these outcomes. We hypothesized that COVID-19 would be independently associated with excess prescription of autonomic medications and increased use of diagnostic head-up tilt testing.

This investigation was necessary for several reasons. Autonomic dysfunction significantly impairs functional capacity, increases the risk of falls and cardiovascular events, and reduces the quality of life. Early recognition and appropriate pharmacological interventions are essential to mitigate these consequences. Furthermore, understanding the evolving demand for autonomic pharmacotherapy in the context of COVID-19 will inform clinicians, health systems, and policymakers, as they adapt to the ongoing challenges of the post-pandemic era.

Here, we report results from the largest population-based analysis to date of post-COVID autonomic drug prescription, encompassing over six million adults and up to two years of follow-up. By presenting detailed hazard ratios and subgroup analyses, we aimed to provide robust, actionable evidence to inform both clinical care and future research in this rapidly evolving field.

## Materials and methods

### Data source and study design

We performed a retrospective, observational, matched cohort study using the National Health Insurance claims database in the Okayama, Hiroshima, Kyoto, Osaka, Hyogo, and Tottori prefectures, which covers 16 million residents and includes detailed information on outpatient and inpatient services, procedures, and drug dispensing records.

### Cohort assembly

Eligible individuals were all diagnosed with COVID‑19 or control group between January 1, 2020, and December 31, 2022. We excluded individuals with any prescription who had been prescribed the outcome drugs or received a head‑up tilt test within 1 year prior to the index to ensure incident outcomes.

### Propensity‑score matching

Propensity-score matching is used in this study to create comparable groups of COVID-19 patients and controls by balancing observed covariates that could influence both the likelihood of COVID-19 infection and the outcome of interest. We calculated propensity scores incorporating age categories, sex, calendar month, comorbidities, and baseline antihypertensive, antidiabetic, and psychotropic medications, which are known risk factors for autonomic dysfunction. Propensity score matching was performed using nearest-neighbor 1:1 matching with replacement. A caliper equal to 0.2, is used. Details of these factors are provided in Supplemental Tables 1 and 2. This method reduces confounding bias by matching individuals with similar baseline characteristics, allowing for a more accurate estimation of the effect of COVID-19 on subsequent prescription rates. In addition to age, sex, and underlying diseases included in the Charlson Comorbidity Index (CCI), medications associated with diseases reported as risk factors were employed as matching variables [7,8].

**Table 1.** Baseline characteristics.

	Total	Control	COVID-19	SMD
	*N* = 6,148,658	*N* = 3,074,329	*N* = 3,074,329	
Female	3,432,268 (55.8%)	1,716,698 (55.8%)	1,715,570 (55.8%)	0.00074
Age category				–0.00066
0–4	405,752 (7%)	202,876 (7%)	202,876 (7%)	
5–9	319,538 (5%)	159,798 (5%)	159,740 (5%)	
10–14	248,620 (4%)	124,342 (4%)	124,278 (4%)	
15–19	238,761 (4%)	119,417 (4%)	119,344 (4%)	
20–24	269,905 (4%)	134,978 (4%)	134,927 (4%)	
25–29	276,768 (5%)	138,404 (5%)	138,364 (5%)	
30–34	293,054 (5%)	146,480 (5%)	146,574 (5%)	
35–39	301,543 (5%)	150,710 (5%)	150,833 (5%)	
40–44	310,837 (5%)	155,334 (5%)	155,503 (5%)	
45–49	366,699 (6%)	183,366 (6%)	183,333 (6%)	
50–54	361,274 (6%)	180,644 (6%)	180,630 (6%)	
55–59	337,012 (5%)	168,495 (5%)	168,517 (5%)	
60–64	325,420 (5%)	162,758 (5%)	162,662 (5%)	
65–69	347,785 (6%)	174,449 (6%)	173,336 (6%)	
70–74	502,049 (8%)	251,744 (8%)	250,305 (8%)	
75–79	432,395 (7%)	216,609 (7%)	215,786 (7%)	
80–84	376,681 (6%)	188,022 (6%)	188,659 (6%)	
≥85	434,565 (7%)	215,903 (7%)	218,662 (7%)	
CCI				–0.00158
0	1,048,759 (17%)	524,615 (17%)	524,144 (17%)	
1	2,034,232 (33%)	1,017,515 (33%)	1,016,717 (33%)	
2–3	1,568,929 (26%)	785,469 (26%)	783,460 (25%)	
4 or over	1,496,738 (24%)	746,730 (24%)	750,008 (24%)	
Comorbidities				
AMI	144,644 (2.4%)	70,873 (2.3%)	73,771 (2.4%)	–0.00622
RD	327,784 (5.3%)	161,748 (5.3%)	166,036 (5.4%)	–0.00281
AIDS	4,574 (0.1%)	2,212 (0.1%)	2,362 (0.1%)	–0.00120
CHF	1,069,328 (17.4%)	533,026 (17.3%)	536,302 (17.4%)	0.00019
CEVD	999,049 (16.2%)	498,846 (16.2%)	500,203 (16.3%)	–0.00384
Dementia	327,150 (5.3%)	163,640 (5.3%)	163,510 (5.3%)	–0.00578
Rheumatoid Disease	296,485 (4.8%)	146,979 (4.8%)	149,506 (4.9%)	–0.00562
Amyloidosis	9,559 (0.2%)	4,366 (0.1%)	5,193 (0.2%)	−0.00683
PD	100,356 (1.6%)	49,049 (1.6%)	51,307 (1.7%)	−0.00580
Diabetes	518,225 (8.4%)	256,712 (8.4%)	261,513 (8.5%)	−0.00562
Medication				
CCB	709,711 (11.5%)	352,197 (11.5%)	357,514 (11.6%)	–0.00541
ACEI/ARB	687,839 (11.2%)	341,566 (11.1%)	346,273 (11.3%)	–0.00486
Hypoglycemic agents	357,067 (5.8%)	175,904 (5.7%)	181,163 (5.9%)	–0.00731
Diuretics	302,953 (4.9%)	148,859 (4.8%)	154,094 (5.0%)	–0.00787
β-blockers	158,231 (2.6%)	77,085 (2.5%)	81,146 (2.6%)	–0.00834
Alfa 1b	125,121 (2.0%)	60,830 (2.0%)	64,291 (2.1%)	–0.00797
Antidepressants	108,058 (1.8%)	52,869 (1.7%)	55,189 (1.8%)	–0.00574
SGLT2i	105,443 (1.7%)	51,274 (1.7%)	54,169 (1.8%)	–0.00725
AntiDementia	94,533 (1.5%)	47,158 (1.5%)	47,375 (1.5%)	–0.00057
Nitrates	60,508 (1.0%)	28,870 (0.9%)	31,638 (1.0%)	–0.00912
DMARD	59,248 (1.0%)	28,816 (0.9%)	30,432 (1.0%)	–0.00538
Anti-parkinson	52,431 (0.9%)	25,398 (0.8%)	27,033 (0.9%)	−0.00578
PDE5i	11,949 (0.2%)	5,760 (0.2%)	6,189 (0.2%)	–0.00317
Alpha 2 ag	10,813 (0.2%)	5,003 (0.2%)	5,810 (0.2%)	–0.00627

SMD: standardized mean difference; CCI: Charlson comorbidity index; AMI: acute myocardial infarction; RD: renal disease; AIDS: acquired immune deficiency syndrome; CHF: congestive heart failure; CEVD: cerebrovascular disease; PD: Parkinson’s disease; CCB: calcium channel blocker; ACEI: angiotensin-converting enzyme inhibitor; ARB: angiotensin II receptor blocker; SGLT2i: sodium–glucose cotransporter-2 inhibitor; DMARD: disease-modifying antirheumatic drug; PDE5i: phosphodiesterase type 5 inhibitor; Alpha 1b: α1-adrenergic receptor blocker; Alpha 2 ag: α2-adrenergic receptor agonist; AntiDementia: anti-dementia medications; Anti-parkinson: anti-Parkinson’s disease drugs; Hypoglycemic agents, including insulin and oral antidiabetic drugs. SMD values less than 0.1 were interpreted as indicating negligible imbalance between groups. Age and CCI were also categorized. Medications were identified on the basis of prescriptions at baseline.

### Exposure and outcomes

During the study period, all cases of SARS-CoV-2 infection in Japan were considered mandatory for reporting illnesses under the Infectious Disease Control Law and were fully reimbursed by public insurance when diagnosed at a medical institution. In this study, cases that were reimbursed by public insurance were considered exposures. The primary composite outcome was the first outpatient prescription of any autonomic dysfunction medication, including midodrine (ATC C01CA17), fludrocortisone (H02AA02), amezinium methylsulfate (C01CA25), or droxidopa (C01CA27), recorded after the index month. The secondary outcomes were individuals who underwent the head-up tilt test. Follow-up was censored by insurance disenrollment or 24 months, whichever occurred first.

### Statistical analysis

Cumulative incidence curves were generated using the Kaplan–Meier estimator. Cox proportional hazards models yielded hazard ratios (HRs) with 95 % confidence intervals (CIs). Subgroup analyses examined modifications by age, sex, Charlson index (four‑tile), medication classes, and comorbidities. Interaction p-values were computed using likelihood‑ratio comparison of models with and without the cross-product term. For the sensitivity analysis, the period was divided into two separate analyses: within one year and after one year.

All tests were two-tailed with α = 0.05. Analyses were performed using Stata 18 software (StataCorp, TX, USA). The study was approved by the institutional review board with a waiver of informed consent due to de-identification.

### Ethical consideration

The Epidemiological Research Committee of Hiroshima University reviewed and approved the research protocol (approval number E2022-0024-01), and all work was conducted with the formal approval of the committees. The committee waived the requirement for informed consent as the data was collected anonymously. The study was conducted in accordance with the principles outlined in the Declaration of Helsinki.

## Results

### Cohort characteristics

Baseline characteristics of the study population (3074329 pairs) are shown in Table 1. The population comprised 55.8% females in both groups. Age categories were evenly distributed across the exposed and unexposed groups, with the largest proportions in the 70–74 years (8%) categories. The CCI showed similar distributions in both groups, with 33% having a CCI of 1, 26% having a CCI of 2–3, and 24% having a CCI ≥ 4. Comorbidities, such as chronic heart failure (17.4%) and cerebrovascular disease (16.2%), were prevalent. Medication use was also comparable between the groups, with calcium channel blockers (11.5%) and ACE inhibitors/ARBs (11.2%) being the most common. The standardized mean differences for all variables were < 1%, indicating a well-balanced group.

### Composite and primary outcome

**Figure 1.**
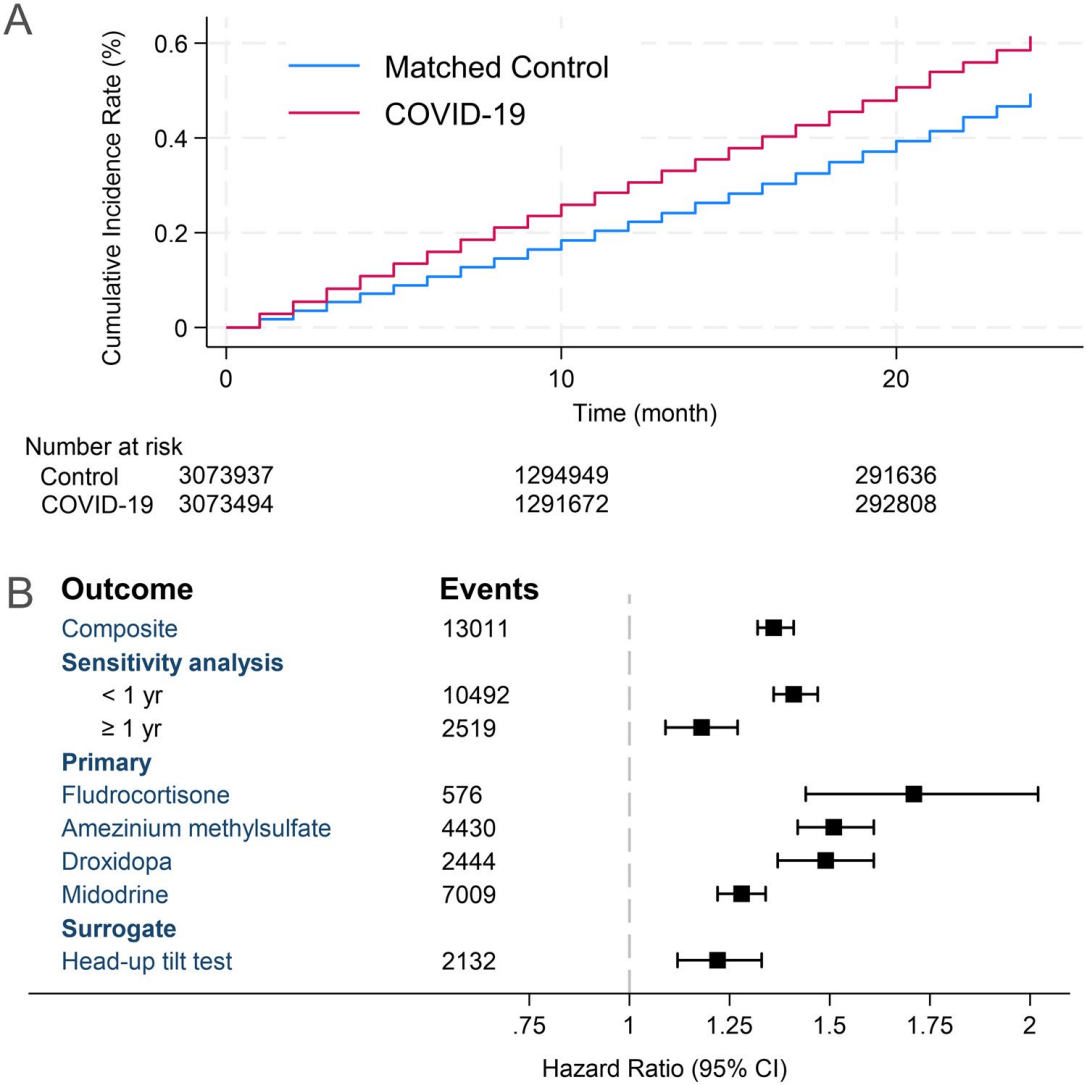
Kaplan Meier Curve and Hazard ratios for outcomes. (A) Cumulative incidence for composite outcome; (B) Hazard ratio for composite, primary, and surrogate outcomes with number of events. The results of the sensitivity analysis are also shown for an interval of less than one year and after one year.

Over 58.1 million person‑months (median follow-up, 8 months), we observed 13,011 composite events overall. The overall HR was 1.36 (95 % CI 1.32–1.41). COVID‑19 infection was also associated with increased initiation of each medication: midodrine HR 1.28 (95 % CI 1.22–1.34; 7,009 events), fludrocortisone HR 1.71 (1.44–2.02; 1,242 events), amezinium methylsulfate HR 1.51 (1.42–1.61; 2,107 events) and droxidopa HR 1.49 (1.37–1.61; 2,653 events) (Figure 1, Supplementary Table 3).

Sensitivity analysis revealed varying effects based on the duration of follow-up. For patients with less than 1 year of follow-up, the adjusted hazard ratio was 1.41 (95% CI: 1.36–1.47), indicating a 41% increased risk. In contrast, patients followed for more than 1 year showed a lower but still significant increased risk, with an adjusted hazard ratio of 1.18 (95% CI: 1.09–1.27). The surrogate outcome, assessed using the head-up tilt test, demonstrated an adjusted hazard ratio of 1.22 (95% CI: 1.12–1.33), suggesting a 22% increased risk associated with this diagnostic procedure.

### Subgroup analysis

**Figure 2.**
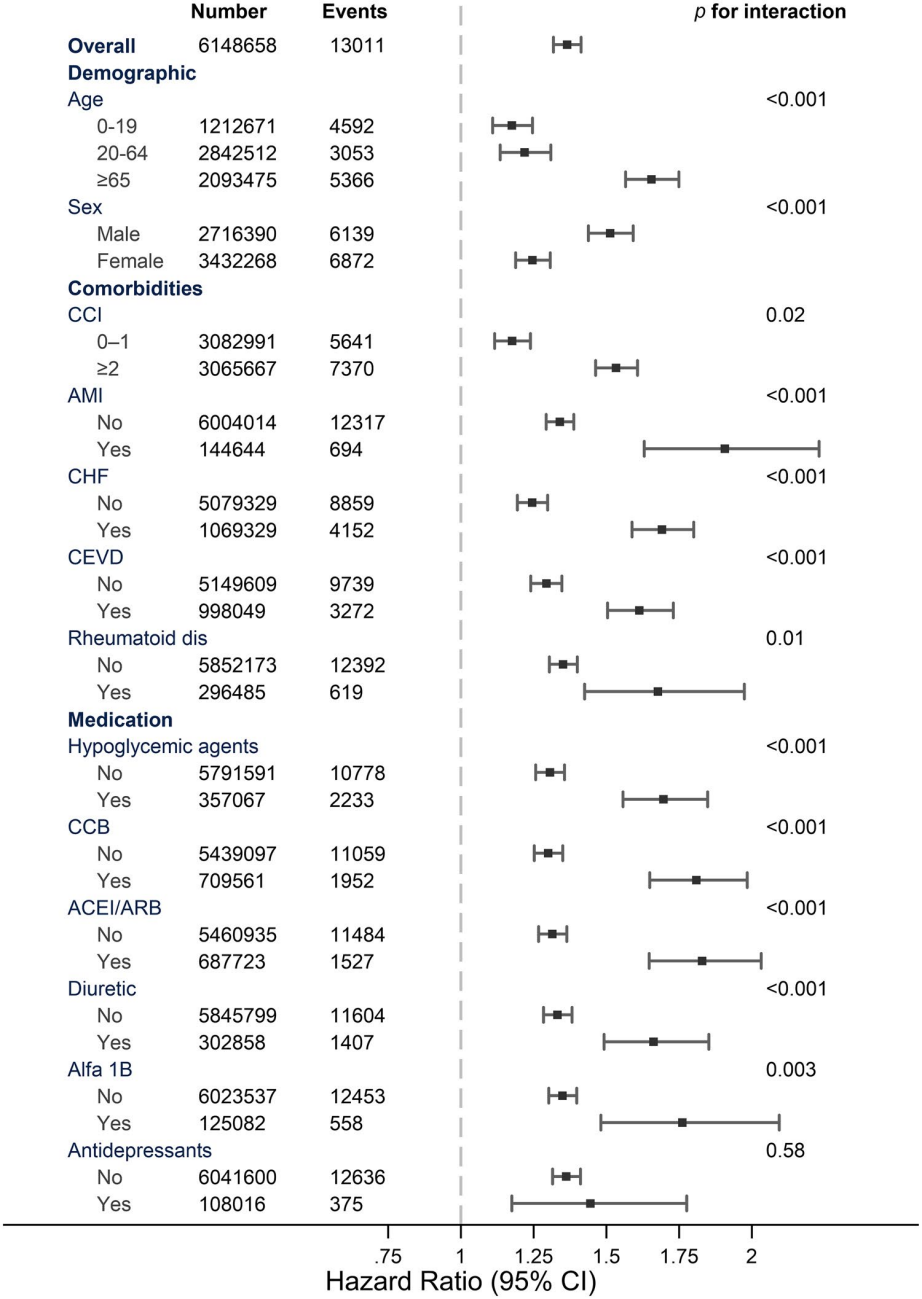
Subgroup analysis with likelihood ratio test. CCI: Charlson comorbidity index; AMI: acute myocardial infarction; RD: renal disease; AIDS: acquired immune deficiency syndrome; CHF: congestive heart failure; CEVD: cerebrovascular disease; CCB: calcium channel blocker; ACEI: angiotensin-converting enzyme inhibitor; ARB: angiotensin II receptor blocker; alpha 1b: α1-adrenergic receptor blocker.

Subgroup analysis revealed heterogeneity in the association between COVID-19 and subsequent outcomes across the different patient characteristics (Figure 2, Supplementary Table 4). Age emerged as a crucial factor, with older individuals (≥65 years) showing a markedly higher risk (HR 1.66, 95% CI 1.57–1.75) compared to younger age groups. Males exhibited a stronger association (HR 1.51, 95% CI 1.44–1.59) than females. Patients with higher comorbidity burden (Charlson index ≥2) demonstrated an increased risk (HR 1.53, 95% CI 1.46–1.61). Among the comorbid conditions, acute myocardial infarction (HR 1.91, 95% CI 1.63–2.23) and chronic heart failure (HR 1.69, 95% CI 1.59–1.80) showed notably higher risks. Medication exposure analysis revealed stronger associations for patients using ACE inhibitors/ARBs (HR 1.83, 95% CI 1.65–2.03) and calcium channel blockers (HR 1.81, 95% CI 1.65–1.98).

## Discussion

In the largest study to date examining pharmacological proxies of autonomic dysfunction after COVID‑19, we found a 36 % relative increase in prescriptions and a modest absolute risk increment of 0.81 events per 10,000 person‑years over two years. Importantly, the signal persisted beyond one year, underscoring the chronicity of the post-acute sequelae.

Our findings extend previous mechanistic observations by providing population-level data on clinically actionable endpoints. Whereas previous studies relied predominantly on symptom surveys or small autonomic testing cohorts [9], we leveraged pharmacy claims, a hard-utilization metric less susceptible to reporting bias. The drug‑specific gradient we observed—largest for fludrocortisone, smallest for midodrine—may reflect differential physician preference or underlying pathophysiology; fludrocortisone is often reserved for more severe volume‑depleted orthostatic hypotension, potentially signifying more profound dysautonomia in post-COVID patients.

Observational cohorts for long-covid patients report symptomatic autonomic dysfunction in 38.5–66 % of post-COVID- patients within the first year[3,10]. These prevalence estimates are numerically far higher than the 0.28 % two-year cumulative incidence of prescriptions observed in our study, reflecting differences in case definitions (symptoms vs. medication initiation) and the proportion of patients managed non-pharmacologically. The results were controversial, with reports ranging from no effect on autonomic neuropathy [11], and another study examining USA veterans found a 1.5-fold increased risk over 3 years [1]. Our study examined young adults and minors together, and found a significantly increased risk in all groups.

In addition, past studies have reported that antihypertensive medications and rheumatic diseases are at risk, and in this study, we found symptoms including a significant interaction. Previous studies have also reported that women [12], the elderly, neurological diseases, diabetes [7], autoimmune diseases, antihypertensive medications, hypoglycemic medications, and vasodilators are at risk [13]. In the present study, an interaction with COVID-19 was observed in patients over 65 years of age receiving antihypertensive drugs. In addition, an increased risk of cognitive function (HR 1.54 CI 1.20–1.97) [14], cardiovascular risk (HR 2.7 CI 1.5–4.83) [15], and heart failure (1.30 CI 1.09–1.55) [16] have been reported. The increased risks observed in this study, including significant interactions in patients with comorbidities, such as those receiving antihypertensive medications, suggest that COVID-19 may exacerbate underlying conditions. Notably, although orthostatic hypotension has been reported as a risk factor for certain diseases, our findings suggest that it may act in a mutually reinforcing manner with both COVID-19 infection and underlying comorbidities. This interaction could potentially amplify the severity or progression of background diseases through synergistic mechanisms.

In our study, utilization increased after COVID-19 was diagnosed for all drugs. The actual status and efficacy of therapeutic agents for POTS and orthostatic hypotension after the COVID-19 pandemic have not been fully verified [6]. In fact, SRs conducted after the COVID-19 pandemic have estimated that treatment with midodrine and ivabradine is effective for POTS. However, the treatment options for POTS during COVID-19 have not been fully explored [17]. In addition, while midodrine and droxidopa have been recommended for orthostatic hypotension [18], the actual treatment status related to COVID-19 is not clear. This study found that midodrine was the most commonly used medication in Japan, followed by amezinium methylsulfate, droxidopa, and fludrocortisone. These factors are because in Japan, insurance coverage for drugs indicated for orthostatic hypotension and POTS is limited to midodrine and amezinium methylsulfate, while droxidopa is restricted to the treatment of Shy-Drager, amyloid neuropathy, Parkinson’s disease, and hemodialysis patients. Fludrocortisone is approved by the insurance for use in Addison’s disease [19]. The limited availability of drugs is thought to have influenced these realities. Although beta-blockers and other agents have been recommended for POTS, they were not used as outcomes in this study because of the large number of indications rather than POTS. The use of ivabradine is also restricted in Japan to patients with chronic heart failure who are in sinus rhythm and whose resting heart rate is 75 beats/min or higher at the start of treatment, and who are receiving standard treatment for chronic heart failure [19]. The number of tests for head-up tilt, which was used as the surrogate outcome, was relatively small compared to the frequency of prescriptions, and it was assumed that many patients may have been diagnosed and treated with simple tests and other procedures.

Multiple nonmutually exclusive mechanisms may underlie this association. Autopsy studies have demonstrated viral RNA and immune infiltrates within the nucleus tractus solitarius, sympathetic chain, and dorsal vagal complex, suggesting direct neuronal involvement [20]. Autoantibodies against β‑adrenergic and muscarinic receptors have also been detected months after infection, indicating molecular mimicry‑driven autoimmunity [21]. Persistent microvascular inflammation and hypoperfusion may further destabilize the baroreflex loops. The interaction with the renin-angiotensin system blockade aligns with the hypotheses that SARS‑CoV‑2 mediated ACE2 dysregulation alters autonomic tone.

Our study has several notable strengths: (i) a large sample size that ensures robust statistical power; (ii) a rigorous new-user design that mitigates immortal-time bias; (iii) comprehensive adjustment for a wide array of confounders through propensity score matching; and (iv) consistent results in sensitivity analyses. However, certain limitations of this study merit consideration. The claims data lacked detailed information on symptom severity, lying-to-standing blood pressure values, and precise drug adherence. There is a possibility of misclassification of the COVID-19 status, although it is likely non-differential. While we adjusted for measured covariates, residual confounding from unmeasured factors (e.g. health-seeking behavior) cannot be entirely ruled out. Finally, the generalizability of our findings beyond the Japanese healthcare context should be evaluated in other settings.

Clinically, our results advocate routine inquiry into orthostatic symptoms during the follow‑up of COVID‑19 survivors, particularly those with cardiometabolic comorbidities or on RAS blockers. Early tilt testing and where indicated, pharmacotherapy may mitigate functional decline. At the public health level, projected drug demand should inform supply chain planning and guideline development for post‑COVID clinics.

## Conclusion

COVID-19 infection shows significant association with increased autonomic dysfunction pharmacotherapy initiation, with a 36% rise in prescriptions over 8 months. Among COVID-19 survivors, there was a 71% increase in fludrocortisone prescriptions, indicating severe cases of orthostatic hypotension, while midodrine was the most commonly prescribed medication. Risk was higher in older adults, males, and patients with higher comorbidity burdens. Acute myocardial infarction, chronic heart failure, and medications like ACE inhibitors/ARBs and calcium channel blockers increased risk, suggesting COVID-19 interaction with cardiometabolic conditions worsens autonomic dysfunction.

Based on these results, the study recommends routine screening for orthostatic symptoms during follow-up care of COVID-19 survivors, especially for older adults, males, and those with cardiometabolic conditions or on RAS blockers. Healthcare systems must prepare for increased demand for autonomic dysfunction services post-pandemic, guiding supply management and post-COVID clinic guidelines.

## Supplementary Material

Supplemental Material

## Data Availability

Data will be made available on reasonable request from corresponding author, DM. The data are not publicly available due to restrictions, which were used under license for this study. The data that support the findings of this study are available from Japan’s Ministry of Health, Labor, and Welfare (MHLW application number 1502). Data are available at https://www.mhlw.go.jp/content/12400000/001158704.pdf (in Japanese) with the permission of MHLW.
