# Supplementary material for: Impact of COVID‑19 infection on subsequent prescriptions of autonomic dysfunction pharmacotherapy: a nationwide propensity‑score‑matched Cohort study in Japan
Source: Ann Med. 2026 Jan 20;58(1):2618323. doi: 10.1080/07853890.2026.2618323 (PMC12825585; doi:10.1080/07853890.2026.2618323)
Supplement: Supplemental Material [file IANN_A_2618323_SM5110.pdf]

Supplementary table 1: Definition of Covariates for Comorbidities with ICD-10 codes

| Comorbidities | ICD-10 |
|---|---|
| Myocardial infarction | I21.x, I22.x, I25.2 |
| Congestive heart failure | I09.9, I11.0, I13.0, I13.2, I25.5, I42.0, I42.5−I42.9, I43.x, I50.x, P29.0 |
| Cerebrovascular disease | G45.x, G46.x, H34.0, I60.x−I69.x |
| Dementia | F00.x−F03.x, F05.1, G30.x, G31.1 |
| Rheumatic disease | M05.x, M06.x, M31.5, M32.x−M34.x, M35.1, M35.3, M36.0 |
| Diabetes | E10.x−E14.x |
| Renal disease | I12.0, I13.1, N03.2−N03.7, N05.2−N05.7, N18.x, N19.x, N25.0, Z49.0− Z49.2, Z94.0, Z99.2 |
| AIDS/HIV | B20.x−B22.x, B24.x |
| Amyloidosis | E85.X |
| Parkinson’s disease | G20.X |

AIDS: acquired immune deficiency syndrome; HIV: Human Immunodeficiency Virus.

Supplementary table 2: Definition of Covariates for Drug category

| Category | Generic drug names |
|---|---|
| AntiDementia | donepezil; rivastigmine; galantamine; memantine |
| SGLT2i | dapagliflozin; canagliflozin; empagliflozin; ipragliflozin; luseogliflozin; tofogliflozin |
| ACEi & ARBs | enalapril; captopril; lisinopril; perindopril; trandolapril; imidapril; delapril; ramipril; losartan; valsartan; telmisartan; candesartan; olmesartan; azilsartan; irbesartan |
| CCB | amlodipine; nifedipine; azelnidipine; benidipine; nilvadipine; nicardipine; manidipine; barnidipine; cilnidipine |
| Antidepressants | duloxetine; venlafaxine; milnacipran; mirtazapine; imipramine; clomipramine; trimipramine; lofepramine; amitriptyline; nortriptyline; dosulepin; amoxapine; maprotiline; mianserin; setiptiline; vortioxetine |
| Hypoglycemic agents | metformin; buformin; pioglitazone; glibenclamide; gliclazide; glimepiride; acetohexamide; glyclopyramide; repaglinide; nateglinide; mitiglinide; sitagliptin; vildagliptin; saxagliptin; alogliptin; linagliptin; teneligliptin; trelagliptin; anagliptin; omarigliptin; exenatide; liraglutide; lixisenatide; dulaglutide; semaglutide; acarbose; miglitol; voglibose |
| Diuretics | hydrochlorothiazide; indapamide; trichlormethiazide; furosemide; torasemide; azosemide |
| Nitrates | nitroglycerin; isosorbide dinitrate; isosorbide mononitrate; nicorandil |
| PDE5i | sildenafil; tadalafil; vardenafil |
| Alpha 1b | prazosin; doxazosin; urapidil; tamsulosin; terazosin; naftopidil; silodosin |
| Alpha 2ag | methyldopa; clonidine; tizanidine |
| β-blockers | pindolol; propranolol; nadolol; carteolol; metoprolol; atenolol; bisoprolol; esmolol; landiolol; bufetolol; alprenolol; arotinolol |
| Anti-Parkinson | trihexyphenidyl; biperiden; methixene; procyclidine; amantadine; levodopa; carbidopa ; levodopa; benserazide ; levodopa; entacapone ; carbidopa ; levodopa; bromocriptine; pergolide; dihydroergocryptine; ropinirole; pramipexole; cabergoline; apomorphine; rotigotine; selegiline; rasagiline; safinamide; tolcapone; entacapone; opicapone; istradefylline |
| DMARD | gold sodium thiomalate; penicillamine; bucillamine; iguratimod; sulfasalazine; actarit; leflunomide; tacrolimus; azathioprine; methotrexate; mizoribine; tofacitinib; upadacitinib; filgotinib; peficitinib; abatacept; etanercept; infliximab; adalimumab; certolizumab; golimumab; sarilumab; denosumab; baricitinib; tocilizumab |

ARB, angiotensin II receptor blocker; ACE I, angiotensin-converting-enzyme inhibitor; CCB, calcium-channel blocker; DMARD, disease-modifying antirheumatic drug; SGLT2, sodium–glucose cotransporter-2 inhibitor; Alpha 1b, α1-adrenergic receptor blocker; Alpha 2 ag, α2-adrenergic receptor agonist.

Supplementary table 3: Main and sensitivity analysis with primary and surrogate outcomes

| Outcome | Events | HR (95% CI) |
|---|---|---|
| Composite outcome | 13011 | 1.36 (1.32−1.41) |
| Sensitivity analysis | | |
| < 1yr | 10492 | 1.41 (1.36 – 1.47) |
| > 1yr | 2519 | 1.18 (1.09 – 1.27) |
| Primary outcome | | |
| Fludrocortisone | 576 | 1.71 (1.44−2.02) |
| Amezinium methylsulphate | 4430 | 1.51 (1.42−1.61) |
| Droxidopa | 2444 | 1.49 (1.37−1.61) |
| Midodrine | 7009 | 1.28 (1.22−1.34) |
| Surrogate outcome | | |
| Head-up tilt test | 2132 | 1.22 (1.12−1.33) |

Supplementary table 4: Subgroup analysis

| Category | Number | Events | HR (95 % CI) | PI |
|---|---|---|---|---|
| Overall | 6148658 | 13011 | 1.36 (1.32 – 1.41) | |
| Age | | | | |
| 0 – 19 | 1212671 | 4592 | 1.18 (1.11 – 1.25) | < 0.001 |
| 20 – 64 | 2842512 | 3053 | 1.22 (1.13 – 1.31) | |
| ≥ 65 | 2093475 | 5366 | 1.66 (1.57 – 1.75) | |
| Sex | | | | |
| Male | 2716390 | 6139 | 1.51 (1.44 – 1.59) | < 0.001 |
| Female | 3432268 | 6872 | 1.25 (1.19 – 1.31) | |
| Charlson comorbidity index | | | | |
| 0 – 1 | 3082991 | 5641 | 1.18 (1.12 – 1.24) | < 0.001 |
| ≥ 2 | 3065667 | 7370 | 1.53 (1.46 – 1.61) | |
| Comorbid conditions | | | | |
| AMI - No | 6002846 | 12317 | 1.34 (1.29 – 1.39) | < 0.001 |
| AMI - Yes | 144644 | 694 | 1.91 (1.63 – 2.23) | |
| CHF - No | 5078464 | 8859 | 1.24 (1.19 – 1.30) | < 0.001 |
| CHF - Yes | 1069328 | 4152 | 1.69 (1.59 – 1.80) | |
| CEVD - No | 5149609 | 9739 | 1.29 (1.24 – 1.35) | < 0.001 |
| CEVD - Yes | 999049 | 3272 | 1.61 (1.50 – 1.73) | |
| RD – No | 5820874 | 9946 | 1.25 (1.20 – 1.30) | < 0.001 |
| RD - Yes | 327784 | 3065 | 1.87 (1.74 – 2.01) | |
| Dementia - No | 5821508 | 11546 | 1.37 (1.32 – 1.42) | 0.50 |
| Dementia - Yes | 327150 | 1465 | 1.41 (1.28 – 1.57) | |
| Rheumatoid disease - No | 5852173 | 12392 | 1.35 (1.30 – 1.40) | 0.010 |
| Rheumatoid disease - Yes | 296485 | 619 | 1.68 (1.43 – 1.97) | |
| AIDS – No | 6144084 | 12997 | 1.36 (1.32 – 1.41) | 0.13 |
| AIDS – Yes | 4574 | 14 | 3.40 (0.95 – 12.2) | |
| Medication exposure | | | | |
| Hypoglycemic - No | 5790447 | 11766 | 1.34 (1.29 – 1.39) | < 0.001 |
| Hypoglycemic – Yes | 356984 | 1245 | 1.66 (1.48 – 1.87) | |
| ACE I/ARB - No | 5460819 | 11484 | 1.31 (1.27 – 1.36) | < 0.001 |
| ACE I/ARB - Yes | 687839 | 1527 | 1.83 (1.65 – 2.03) | |
| CCB - No | 5438947 | 11059 | 1.30 (1.25 – 1.35) | < 0.001 |
| CCB - Yes | 709711 | 1952 | 1.81 (1.65 – 1.98) | |
| Diuretic - No | 5845705 | 11604 | 1.33 (1.28 – 1.38) | < 0.001 |
| Diuretic - Yes | 302953 | 1407 | 1.66 (1.49 – 1.85) | |
| SGLT2 inhibitor - No | 6043215 | 12849 | 1.36 (1.31 – 1.41) | 0.072 |
| SGLT2 inhibitor - Yes | 105443 | 162 | 1.83 (1.32 – 2.53) | |
| α1-blocker - No | 6023537 | 12453 | 1.35 (1.30 – 1.40) | 0.003 |
| α1-blocker - Yes | 125121 | 558 | 1.76 (1.48 – 2.09) | |
| DMARD - No | 6089417 | 12917 | 1.36 (1.32 – 1.41) | 0.23 |
| DMARD - Yes | 59241 | 94 | 1.76 (1.15 – 2.68) | |
| Anti-Parkinson - No | 6096227 | 12089 | 1.38 (1.33 – 1.43) | 0.002 |
| Anti-Parkinson - Yes | 52431 | 922 | 1.16 (1.02 – 1.32) | |
| Anti-Depressants- No | 6041600 | 12636 | 1.36 (1.31 – 1.41) | 0.58 |
| Anti- Depressants - Yes | 108016 | 375 | 1.44 (1.18 – 1.78) | |

AMI, acute myocardial infarction; ARB, angiotensin II receptor blocker; ACE I, angiotensin-converting-enzyme inhibitor; CCB, calcium-channel blocker; CEVD, cerebrovascular disease; CHF, chronic heart failure; CPD, chronic pulmonary disease; DMARD, disease-modifying antirheumatic drug; SGLT2, sodium–glucose cotransporter-2 inhibitor; PI, p for interaction.
